# Changes in Urinary Arsenic Methylation Profiles in a 15-Year Interval after Cessation of Arsenic Ingestion in Southwest Taiwan

**DOI:** 10.1289/ehp.0900560

**Published:** 2009-07-29

**Authors:** Yung-Kai Huang, Ya-Li Huang, Yu-Mei Hsueh, Jimmy Tse-Jen Wang, Mo-Hsiung Yang, Chien-Jen Chen

**Affiliations:** 1 Graduate Institute of Medical Sciences, College of Medicine Taipei Medical University, Taipei, Taiwan; 2 Department of Public Health, School of Medicine, Taipei Medical University, Taipei, Taiwan; 3 School of Public Health, Taipei Medical University, Taipei, Taiwan; 4 Department of Emergency Medicine, Taipei Medical University Hospital, Taipei, Taiwan; 5 Department of Biomedical Engineering and Environmental Sciences, National Tsing-Hua University, Hsinchu, Taiwan; 6 Genomics Research Center, Academia Sinica, Taipei, Taiwan

**Keywords:** arsenic methylation, cumulative arsenic exposure, drinking water

## Abstract

**Background:**

Inorganic arsenic (iAs) is carcinogenic to humans. Methylated metabolites of arsenic (As) found in the urine could serve as potential tools for screening and early detection of cancer in populations exposed to As. Relatively little information is available regarding changes in As methylation profiles after cessation of As exposure.

**Objective:**

We examined the changes in urinary arsenic (uAs) species profiles over 15 years in a cancer-free population that has ceased heavy and prolonged ingestion of As.

**Methods:**

In 1989, a cohort study was carried out with 1,081 adults who resided in three villages in southwestern Taiwan where arseniasis was hyperendemic. After 15 years of follow-up, a subgroup of 205 cancer-free participants had completed all interviews and had uAs methylation data available. We used this group in our statistical analysis. Arsenic species were measured by high-performance liquid chromatography-hydride generation-atomic absorption spectrometry.

**Results:**

We compared the initial analyses from 1989 with those performed 15 years later and found that the average differences for the proportion of urinary iAs, monomethylarsonic acid (MMA^V^), and dimethylarsinic acid (DMA^V^) were −4.90%, −6.80%, and 11.69%, respectively. The elderly and those residents with longer periods of consuming high-As artesian well water exhibited greater changes (decreases) in %MMA^V^.

**Conclusion:**

The As methylation profiles indicate increased efficiency in As metabolism in residents after cessation of long-term exposure to high-level As. Moreover, the decreased %MMA^V^ was more pronounced in the elderly cancer-free subcohort subjects.

Arsenic is often present in water as inorganic arsenic (iAs). Although commonly found throughout nature, iAs is a human carcinogen (International Agency for Research on Cancer 1980). Chronic As exposure has been associated with a large number of other consequences, including effects on neurologic, reproductive, developmental, genotoxic, and immunologic systems [Agency for Toxic Substances and Disease Registry (ATSDR) 2000].

Blood, urine, hair, and nails are used to assess As exposure and internal As levels in humans. Arsenic is rapidly metabolized after ingestion of iAs from drinking water and is excreted mainly through the urine in humans and most laboratory animals (Vahter 1999, 2000). The urinary arsenic (uAs) level or the sum of As metabolites reflects the absorbed dose of iAs on an individual level, which provides a better quantitative estimate of recently absorbed As. Furthermore, it is a useful marker for ongoing ingestion of As because uAs methylation indices have been found to be fairly stable for 8–10 months (Steinmaus et al. 2005).

Blood As levels are not as reliable of an indicator for monitoring chronic As exposure in humans (ATSDR 2000) because As is rapidly cleared from the blood in most animals (Marafante et al. 1982; Vahter and Norin 1980; Yamauchi and Yamamura 1985). Arsenic tends to accumulate in hair and nails, but these samples may yield less accurate results because of absorbed exogenous As contamination on external surfaces (ATSDR 2000; Hindmarsh 2002).

Evaluation of As methylation efficiency is primarily based on quantifying the relative amounts of the different metabolites in urine. After iAs ingestion, approximately 60–90% of the exposure dosage is excreted in mammalian urine that consists of 10–30% As, 10–20% monomethylarsonic acid (MMA^V^), and 60–80% dimethylarsinic acid (DMA^V^). The process of As methylation is considered a detoxification mechanism because the major methylated metabolites, such as MMA^V^ and DMA^V^, are more readily excreted and less toxic than is iAs. However, recent studies have shown that higher urinary %MMA^V^ is related to the risk of skin and bladder cancers as well as cardiovascular disease (Ahsan et al. 2007; Chen et al. 2003a, 2003b; Hsueh et al. 1997; Huang et al. 2007, 2008; Pu et al. 2007; Steinmaus et al. 2006; Tseng et al. 2005; Yu et al. 2000).

After chronic high-level As exposure, it may take substantial time to excrete the As after cessation of ingestion (Dewar and Lenihan 1956). Our previous studies showed that individuals with higher cumulative exposure to iAs in the past had higher levels of urinary MMA^V^ and DMA^V^, or %MMA^V^ (Hsueh et al. 1998; Huang et al. 2007). A recent animal study found that after subchronic exposure to As in mice, monomethylarsonate preferentially accumulated in the kidney, whereas iAs and dimethylarsonate accumulated in the bladder (Kenyon et al. 2008). These data imply that the uAs species profiles might be an indicator of chronic high-level exposure to As in the past.

Endemic blackfoot disease (BFD) in southern Taiwan and the switch from well water to a tap-water system provides a unique opportunity for determining the consequences of chronic As exposure and subsequent cessation of As ingestion. Residents from the BFD area had used artesian well water for more than 20 years when they were recruited in 1989 for the cohort to participate in the present study. This is the first study to investigate the effects of long-term As ingestion on the body after cessation of chronic As exposure through evaluation of the As methylation indices. The purpose of this study is to examine the changes in uAs species profiles over 15 years (1989–2004) in cancer-free residents in the BFD area. The influencing factors were also assessed in this study.

## Methods

### Study area and the study cohort

The original study cohort has been described in detail in our previous study (Chen et al. 1995). The present study was carried out in villages of Chayi County that have the highest frequency of BFD in Taiwan, with prevalence as high as 13.6%, 9.6%, and 10.3% in the villages of Homei, Fuhsing, and Hsinming, respectively (Wu et al. 1961). Although a tap-water supply system was implemented in the early 1960s, tap-water use remained low until the early 1970s. However, by the mid-1970s artesian well water was no longer used in this area for drinking and cooking.

**Figure 1:**
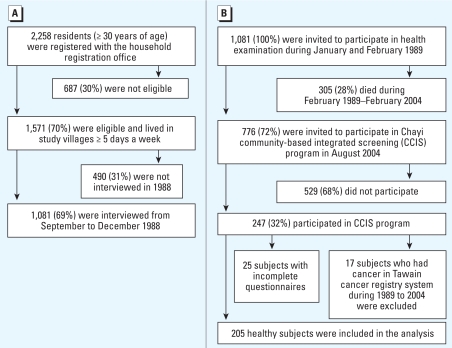
Schematic overview of the recruitment of study subjects from three BFD-hyperendemic villages in southwestern Taiwan: 1989 (*A*) and 2004 (*B*).

Residents ≥ 30 years of age (*n* = 2,258) were registered with the local household registration offices. Seventy percent of residents were eligible for our previous study if they were residing at least 5 days per week in the study villages. Sixty-nine percent (1,081 of 1,571) of eligible residents provided informed consent and became part of the original study cohort. Home interviews with each participant were conducted between September 1988 and December 1988. Participants were then invited, on a voluntary basis, to undergo a health examination, including the collection of a urine sample in January and February 1989 (Figure 1A). The Institutional Review Board of National Taiwan University approved our previous study.

In August 2004, the original cohort residents from the 1989 study were invited to participate in the Chayi Community Based Integrated Screening (CCIS) program. The CCIS program is an integrated model of community-based mass screening and was conducted between 2002 and 2007 in 17 villages and townships in Chayi County. The flowchart of the recruited subcohort is shown in Figure 1B. Of the 1,081 residents from the original cohort, 72% (776) were invited by postcard to participate in the CCIS program. Thirty-two percent (247 of 776) of these invitees participated in the CCIS program in August 2004 and provided informed consent. Each participant’s unique national identification number was used to link to the computerized National Cancer Registry in Taiwan for the purpose of identifying diagnosed cancer cases between 1989 and 2003. After excluding 17 subjects who had developed cancer from 1989 to 2004, and another 25 subjects who provided incomplete questionnaires, a subgroup of 205 cancer-free residents of the original study cohort was available for the data analysis. The Institutional Review Board of Taipei Medical University approved the recruitment of the subcohort in 2004.

### Questionnaire interview and determination of As exposure

The questionnaire interview and the determination of As exposure of subjects residing in the endemic BFD villages of southwestern Taiwan have been reported previously (Chen et al. 1995). Trained public health nurses carried out the standardized personal interviews based on a structured questionnaire between September 1988 and December 1988. In August 2004, subcohort participants were interviewed similarly through the same questionnaire again.

To determine the chronic As exposure indices of residents living in different locales with varying levels of iAs in well water, our questionnaire included residential history (e.g., villages of residence and duration) in addition to duration and source of water consumption. Arsenic levels in artesian well water were obtained from previous research of 155 well-water samples from 42 villages with endemic BFD (Kuo 1964). The cumulative As exposure (CAE) index was used in our analysis to represent a cumulative dose of As in each study subject to reflect individual changes in residence, differing well-water As concentrations, and varying length of water consumption. The CAE was expressed in milligrams per liter-years (mg/L-year). It can then be calculated by the following formula:


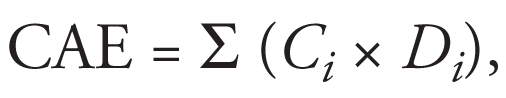


where *C_i_* is the median As concentration of well water (milligrams per liter) in the village where the subject lived, and *D_i_* is the duration in years of well-water consumption while residing in the village. The average As concentration can then be calculated by the formula


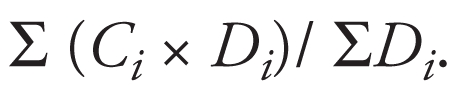


The average arsenic As concentration and the CAE could not be calculated with precision for 54 study subjects (26.3%) who had returned or moved into the study villages after they lived in other areas for several years. CAE and the average As concentration for a given subject were considered to be unknown if the median As concentration of any village where the subject had resided during his or her lifetime was not available.

The cigarette smoking information was extracted from the questionnaires from both study periods. Residents were defined as nonsmokers if they denied ever smoking in their lives. Residents who smoked and never quit between the two study periods were defined as smokers. Those who quit cigarette smoking before the 1989 interview or who had quit between the 1989 and 2004 interviews were defined as former smokers. Incident smokers are those who were originally classified as nonsmokers at the 1989 interview but had become smokers by the 2004 interview. Recurrent smokers were those who were former smokers at the 1989 interview but had become smokers again by the 2004 interview. A total of 5 participants were incident (1) or recurrent smokers (4) and were excluded from the analysis.

### Determination of uAs species

A high-performance liquid chromatography/hydride generator and an atomic absorption spectrometer were used to measure urinary arsenite (As^III^), arsenate (As^V^), MMA^V^, and DMA^V^. Analytical methods for uAs species determinations are reported in our previous study (Hsueh et al. 1998). The quality assurance and control of the laboratory protocol in the urine sample measurements were similar in 1989 and 2004.

The As methylation indices are defined as the percentages of respective uAs species (As^III^, As^V^, MMA^V^, and DMA^V^) present in urine samples, and U-Asmet is the sum of iAs and its metabolites (iAs + MMA^V^ + DMA^V^). The primary methylation index (PMI) was defined as the ratio between MMA^V^ and iAs levels, and the secondary methylation index (SMI) was defined as the ratio between DMA^V^ and MMA^V^. To quantify the changes in uAs methylation profiles between 1989 and 2004, the differences were calculated by subtracting the uAs methylation indices of 1989 from those of 2004.

### Statistical analyses

We used chi-square analysis to test for the association of categorical variables and paired *t*-test to compare the uAs indices between 1989 and 2004. Analysis of variance (ANOVA) and Scheffe’s post hoc test were analyzed to compare uAs methylation indices among three or more groups. Multivariate regression analysis was carried out to study the relationship between differences in urinary methylation profiles with respect to age, sex, cigarette smoking status, and chronic As exposure indices.

## Results

In Table 1, we show the distribution of demographic characteristics and lifestyles of the original cohort in 1989 and the subcohort of 205 cancer-free participants in the arseniasis area of southwestern Taiwan in 2004. The cancer-free participants were generally younger and more educated than were the original cohort, and they had lower CAE and shorter duration of consuming high-As artesian well water. Because chronic arsenic exposure increases the risk of cancer (Chen et al. 1985, 1992; Wu et al. 1989), it is logical that the cancer-free participants had lower cumulative exposure to arsenic in the past.

Table 2 presents intraindividual differences in As methylation indices of residents in 1989 and 2004. Generally speaking, samples collected in 2004 had lower %iAs and %MMA^V^ but higher %DMA^V^ and SMI than did the urine samples collected in 1989. This finding suggests an overall increase in As metabolism efficiency during the 15-year period. We found no difference in the urinary PMI of samples collected in 1989 and 2004.

We also provide the correlation coefficients of uAs indices among healthy residents between 1989 and 2004 (Table 3). The percentage of %iAs in 2004 was positively correlated with %MMA^V^ in 1989 (*r* = 0.26, *p* < 0.001). The correlation coefficient of %iAs in 2004 was decreased with a %DMA^V^ increment in 1989 (*r* = −0.16, *p* < 0.05). We found a negative correlation between SMI in 1989 and %iAs in 2004 (*r* = −0.17, *p* < 0.05). Conversely, SMI in 1989 demonstrated a positive correlation with %DMA^V^ in 2004 (*r* = 0.17, *p* < 0.05).

In Table 4, we demonstrate the interindividual difference of uAs species indices stratified by duration of high-As well-water consumption and age at baseline. The elderly (> 50 years) and those with a longer duration of high-As artesian well-water consumption (≥ 21 years) demonstrated significantly smaller changes in %MMA^V^ from 1989 to 2004 than did the younger residents (< 50 years) and subjects who had consumed high-As well water for < 20 years.

**Figure 2:**
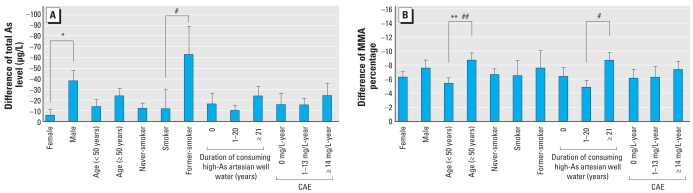
Changes in uAs species indices in 2004 versus 1989 (mean + SE). (*A*) U-Asmet level; (*B*) MMA percentage. *p < 0.05 by multiple regression model, adjusted for age, smoking status, and any one of the As exposure indices. **p < 0.01 by t-test. ^#^*p* < 0.05 by ANOVA and Scheffe’s test. ^##^*p* < 0.05 by multiple regression model, adjusted for sex, smoking status, and any one of the arsenic exposure indices.

Figure 2 depicts the interindividual differences in uAs methylation profiles between 1989 and 2004 (calculated by subtracting methylation indices of 1989 from those of 2004). The differences in As methylation profiles were stratified by age, sex, cigarette smoking status, CAE, and duration of high-As artesian well-water consumption. As shown in Figure 2A, U-Asmet levels dropped significantly more in males than in females (38.60 vs. 5.76 μg/L) and in former smokers than in smokers (62.4 vs. 12.69 μg/L). A multivariate regression model revealed significantly greater changes in U-Asmet in men than in women after adusting for age, cigarette smoking status, and any one of the As exposure indices (duration of high-As artesian well-water consumption, average concentration of As in artesian well water consumed, or CAE). Changes in %MMA^V^ were influenced by age and the duration of high-As artesian well-water consumption (Figure 2B). The older residents (≥ 50 years) showed a significant decrease in %MMA^V^ compared with younger residents (< 50 years; 8.84% vs. 5.46%). The difference among %MMA^V^ in residents who had consumed high-As artesian well water for ≥ 21 years was significantly higher than among residents who had consumed high-As artesian well water for 1–20 years (8.77% vs. 5.95%). In addition, the %MMA^V^ decreased further with increasing age after adjusting for sex, cigarette smoking status, and any one of the As exposure indices.

## Discussion

This is a unique study population with data on determinants of changes in uAs metabolite profiles after cessation of exposure to high- level As water (700 μg/L) for 30–45 years. For the subjects in this study, the intraindividual As methylation profiles appeared to have become more efficient during the 15-year period. Other studies have demonstrated that uAs methylation profiles remained fairly stable for 5 days among an Argentinean population exposed to As at 150–170 μg/L (Concha et al. 2002). Meanwhile, a study in Utah demonstrated that profiles were stable for 8–10 months after exposure to 20 μg/L As (Steinmaus et al. 2005).

Repeated oral exposure of As^V^ (0.5 mg As/kg) in mice had no effects on the uAs methylation profiles (Hughes et al. 2003). Therefore, in humans, other mechanisms may be responsible for changes in As methylation profiles after chronic exposure to As via daily drinking water. The saturation of the As methylation system may in part explain changes in the urinary profiles. In a study on four human volunteers, Buchet et al. (1981) extrapolated that the methylation may begin to become limiting at doses of about 0.2–1 mg/day. Because only limited studies are available with very few subjects, data on the saturation of the methylation system in humans may not be well understood (ATSDR 2000). Nevertheless, we suspect, based on this relatively small subset of previously published data, that the As methylation profiles may be stable if the As exposure is below a certain threshold. Further research is needed to determine the effects of As exposure on human tolerance and consequences of disease burden.

In the present study, we found the subcohort of cancer-free residents to be more efficient in methylating As (Table 2). One possible reason was demonstrated in an animal study by Kenyon et al. (2008), who found that mice preferentially accumulate MMA in the kidney after subchronic exposure to As^V^ in drinking water. Whether cancer-free subjects in this study also preferentially accumulate MMA in the kidney and its potential effect on cancer prevention remains to be seen. Another possible explanation may be related to renal function. Renal function is known to decline with age in humans (Mühlberg and Platt 1999). Renal function data were not available for this study; therefore, we were unable to determine its influence on the methylation profiles.

U-Asmet is a better index for estimating As toxicity (Calderon et al. 1999) than is the analysis of total As in urine because the latter would result in a higher As level (Le et al. 1994) by including nonharmful As forms such as arsenobetaine, arsenocholine, and arsenosugars from seafood. U-Asmet was shown in other studies to be highly correlated with the total As concentration in drinking water (W-TotAs; *r* = 0.86) (Kurttio et al. 1998). As shown in Table 5, U-Asmet increased with W-TotAs in all studies (*r* = 0.56). The greater decrease of U-Asmet in the cancer-free group in this study could be due to the cessation of the exposure.

The U-Asmet:W-TotAs ratios can be used to represent the amount of As accumulated in the body in relation to exposure to chronic and high levels of As from drinking water. If the urinary excretion of As is 100% attributable to the As intake, then the U-Asmet:W-TotAs ratio will trend toward 1.0. The U-Asmet:W-TotAs ratio reported in one study in Inner Mongolia was different from other reported studies, possibly because of differences in the handling of urine samples (samples from this previous study were exposed to 2 M sodium hydroxide and heated at 95°C for 3 hr before the determination of uAs species; Pi et al. 2002). With the exception of the Inner Mongolia study, the U-Asmet:W-TotAs ratios ranged from 0.65 to 1.81 for populations exposed to As > 20 μg/L. The U-Asmet:W-TotAs ratio was < 1 in a Chilean population, which consumed water with As concentrations > 500 μg/L (Chung et al. 2002; Hopenhayn-Rich et al. 1996b). Hopenhayn-Rich et al. (1996a) found that the U-Asmet:W-TotAs ratio was 3.7 in Chile after changing As levels in the drinking water from 600 to 45 μg/L for 2 months. This finding implied that As had accumulated in the Chilean subjects after chronic and heavy exposure to As through drinking water.

The As concentration allowance of public water supplies in Taiwan was changed from 0 μg/L to a new standard of 10 μg/L in 2000. Thus, the U-Asmet:W-TotAs ratios increased from 1.50 (74.86/50) to 5.71 (57.08/10) during the 15-year interval from 1989 to 2004. We cannot exclude the intake of seaweed that may have interfered with U-Asmet levels. However, these residents had never left their townships, and it was reasonable to assume that they had not experienced any dramatic changes in their dietary habits over the course of this study. Our previous study also did not find an association between frequencies of dietary intake of fish, shellfish, and seaweed and the levels of uAs species in subjects who drank tap water. In addition, As methylation patterns were similar before and after refraining from eating seafood for 3 days (Hsueh et al. 2002). Therefore, the U-Asmet:W-TotAs ratio increased, supporting the hypothesis that As accumulates in the body of those individuals who had ever been exposed to chronic and high levels of As from drinking water.

Another interesting finding from the present study was the more pronounced decrease of %MMA^V^ among members of the elderly cancer-free subcohort. The age of the cancer-free subcohort was highly correlated with the duration of artesian well-water consumption (*r* = 0.62, *p* < 0.0001). Cellular As adaptation is a dynamic process that is mediated by redox homeostasis and recycling of *S*-adenosylmethionine, as shown in a recently published *in vitro* study (Coppin et al. 2008). Urinary and skin %DMA^V^ was increased in mice after repeated oral administration of As^V^ (Hughes et al. 2003; Kenyon et al. 2008). We suspect that the As adaptation among the elderly cancer-free subcohort in the present study may play a role in the significant drop in %MMA^V^ and requires further investigation.

The possibility of genetic contribution to As methylation efficiency was also suggested by studies of native women of the Andes excreting low urinary levels of MMA (2.3–3.5%) regardless of drinking water As levels (Concha et al. 1998; Vahter et al. 1995). Polymorphisms of genes related to As methylation may also contribute to the differences in As methylation profiles. Studies have shown that polymorphisms of MMA reductase or As methyltransferase are related to uAs methylation profiles (Marnell et al. 2003; Schmuck et al. 2005; Wood et al. 2006). Recently, polymorphisms of As methyltransferase also have been shown to affect the ratio of MMA to DMA in urine (Fujihara et al. 2008; 2009; Hernandez et al. 2008). Therefore, genetic controls over the regulatory enzymes of As metabolism may partly explain the substantial variations in As methylation efficiency among different ethnicities.

Two novel As species, monomethylarsonous acid (MMA^III^) and dimethylarsinous acid (DMA^III^), were recently identified in urine (Mandal et al. 2001; Valenzuela et al. 2005). These two novel species were thought to be the more toxic intermediates in the biotransformation of ingested iAs (Styblo et al. 2000; Thomas et al. 2001; Vega et al. 2001). The detection of the transient metabolites of MMA^III^ and DMA^III^ depends on the conditions of sample storage and their concentration in the urine, which was beyond the analytical detection at the time of this study in 1989. Thus, it is difficult to use these trivalent As metabolites as a marker for this study. Further investigations focusing on the association between these highly toxic species of As metabolic intermediates and clinical diseases could be potentially meaningful.

Certain limitations of this study should be noted. First, we had a low response rate and limited sample sizes, a high proportion of women, and the past As exposure among the subcohort of 205 cancer-free participants was low. Thus, it may not be possible to generalize or extrapolate the results of this study to other populations. Second, the 15-year interval is too long to exclude other factors that might have influenced the As methylation profiles, including As levels in drinking water and other environmental sources such as seaweed, occupational exposure, and air contamination. Nutritional status and dietary intake may also be uncontrollable factors during the long study interval. Arsenic methylation involves the addition of a methyl group to iAs or MMA. This one-carbon metabolism can be influenced by dietary substances such as cysteine, methionine, folic acid, vitamin B_12_, and choline in food (Ahsan et al. 2007; Chen et al. 2007; Gamble et al. 2005; Schläwicke Engström et al. 2009; Vahter 2007). Other studies have indicated that, under normal conditions, As methylation is not enhanced by supplementation with methyl donors in humans (Buchet et al. 1982) or in animals (Buchet and Lauwerys 1987). Although no data were available on the change in nutrition status, we found that baseline body mass index was not associated with changes in urinary As methylation profiles (data not shown).

In conclusion, the As methylation profiles appeared to become more efficient among subjects after cessation of long-term exposure to high levels of As. Moreover, the decrease of %MMA^V^ was more pronounced among elderly cancer-free subcohort subjects. These results may have implications for As mediation strategies in areas currently exposed to potentially harmful levels of As in drinking water.

## Figures and Tables

**Table 1:** Distribution of demographic and lifestyle characteristics of the cohort and subcohort participants in the arseniasis area of southwestern Taiwan.

Variable	1989 cohort (*n* = 1,081)	2004 followed healthy subcohort (*n* = 205)	Chi-square value (*p*-value)
Age at enrollment [years, *n* (%)]			
30–39	265 (24.51)	67 (32.68)	18.37 (*p* < 0.01)
40–49	262 (24.24)	57 (27.80)	
50–59	365 (33.77)	68 (33.17)	
60–69	158 (14.62)	13 (6.34)	
≥ 70	28 (2.59)	0 (0.00)	
Data unavailable	3 (0.28)	0 (0.00)	

Sex [*n* (%)]			
Female	605 (55.97)	130 (63.41)	3.90 (*p* = 0.05)
Male	476 (44.03)	75 (36.59)	

Education level [*n* (%)]			
Elementary school or less	880 (81.41)	152 (74.15)	6.13 (*p* = 0.01)
Junior high school or higher	198 (18.32)	53 (25.85)	
Data unavailable	3 (0.28)	0 (0.00)	

Body mass index [kg/m^2^, *n* (%)]			
< 24	434 (40.15)	91 (44.39)	0.54 (*p* = 0.76)
24–27	316 (29.23)	60 (29.27)	
≥ 27	192 (17.76)	35 (17.07)	
Data unavailable	139 (12.86)	19 (9.27)	

Cigarette smoking status [*n* (%)]			
Nonsmoker	831 (76.78)	167 (81.46)	1.90 (*p* = 0.17)
Smoker or former smoker	247 (22.85)	38 (18.54)	
Data unavailable	3 (0.28)	0 (0.00)	

Alcohol consumption [*n* (%)]			
Never	938 (86.77)	182 (88.78)	0.49 (*p* = 0.49)
Current and former	140 (12.95)	23 (11.22)	
Data unavailable	3 (0.28)	0 (0.00)	

Chronic As exposure indices (mean ± SE)			
Duration of high-As artesian well-water consumption (year)	21.45 ± 0.42	17.21 ± 0.84	*p* < 0.0001^a^
Average concentration of As in artesian well water consumed (mg/L)	0.59 ± 0.01	0.55 ± 0.03	*p* = 0.16^a^
CAE (mg/L-year)	14.27 ± 0.38	10.73 ± 0.72	*p* < 0.0001^a^

Unavailable data were excluded in the statistical analyses.

a *p*-Value for Student *t*-test.

**Table 2:** Distribution of uAs species indices in 1989 and 2004.

Species indices	Urinary samples				*p*-Value for paired *t*-test
	1989		2004		
	Mean	SE	Mean	SE	
uAs species level (μg/L)					
iAs	4.85	0.30	1.42	0.11	< 0.0001
MMA	9.21	0.65	3.12	0.23	< 0.0001
DMA	60.80	3.61	52.55	2.81	0.06
U-Asmet	74.86	4.10	57.08	2.97	< 0.001
Percentage of uAs species					
%iAs	7.69	0.55	2.79	0.19	< 0.0001
%MMA^V^	12.56	0.56	5.76	0.36	< 0.0001
%DMA^V^	79.75	0.84	91.44	0.43	< 0.0001
PMI	2.74	0.23	4.56	1.40	0.24
SMI	9.86	0.66	49.99	16.22	0.02

**Table 3:** Pearson correlation coefficients of uAs indices between 1989 and 2004 among healthy residents.

	2004					
1989	%iAs	%MMA^V^	%DMA^V^	PMI	SMI	U-Asmet
%iAs	−0.02	−0.02	0.02	0.02	−0.09	0.00
%MMA^V^	0.26#	0.03	−0.14	−0.11	−0.04	−0.09
%DMA^V^	−0.16**	−0.01	0.08	0.07	0.08	0.06
PMI	0.09	0.10	−0.13*	−0.04	0.05	−0.05
SMI	−0.17**	−0.11	0.17**	0.05	0.01	−0.02
U-Asmet	0.12*	0.03	−0.08	−0.06	−0.04	0.07

* 0.05 < *p* < 0.1,

** *p* < 0.05,

# *p* < 0.01.

**Table 4:** Differences or changes in uAs species indices stratified by duration of high-As artesian well- water consumption [years (mean ± SE)] and baseline age.

	< 20 years		≥ 21 years	
Arsenic species	Age < 50 years (*n* = 99)	Age ≥ 50 years (*n* = 17)	Age < 50 years (*n* = 25)	Age ≥ 50 years (*n* = 64)
%iAs	−5.58 ± 1.04	−4.3 ± 1.6	−3.9 ± 1.37	−4.39 ± 0.67
%MMA^V^	−4.90 ± 0.91*	−7.53 ± 1.48	−7.69 ± 2.23	−9.19 ± 1.19*
%DMA^V^	10.48 ± 1.41	11.84 ± 2.31	11.59 ± 3.02	13.58 ± 1.39
PMI	4.05 ± 3.01	−1.75 ± 1.24	0.04 ± 1.59	−0.26 ± 0.47
SMI	54.53 ± 33.65	26.23 ± 8.27	25.23 ± 15.03	28.7 ± 7.46
U-Asmet	−10.66 ± 5.52	−18.44 ± 9.16	−26.28 ± 25.66	−25.28 ± 8.31

* *p* < 0.05 by ANOVA and Scheffe’s test.

**Table 5:** Urinary arsenic methylation profiles in adult populations who were exposed to As through drinking water.

	As concentration (μm/L)				Metabolite distribution (%)			
Country	No.	W-TotAs (mean)	U-Asmet (mean)	U-Asmet:W-TotAs ratio	iAs	MMA	DMA	Reference
Argentina	11	200	261.0	1.31	26.0	2.3	72.0	Vahter et al. 1995
	14	23	27.0	1.17	42.0	3.5	54.0	Vahter et al. 1995
	37	210	233.0^a^	1.11	14.0^a^	3.1^a^	83.0^a^	Concha et al. 1998
	110	210	272.0^a^	1.30	12.0^a^	7.4^a^	80.0^a^	Schläwicke Engström et al. 2007

Chile	122	600	583.0	0.97	18.4	15.0	66.6	Hopenhayn-Rich et al. 1996b
	98	15	61.0	4.07	14.9	10.6	74.5	Hopenhayn-Rich et al. 1996b
	44	750	490.2	0.65	16.8	13.9	69.3	Chung et al. 2002

Mexico	35	408	739.8	1.81	31.8	13.7	54.5	Gonsebatt et al. 1997
	34	30	34.0	1.13	7.7	5.2	87.1	Gonsebatt et al. 1997
	10	17	28.0	1.65	16.4	15.0	67.1	Meza et al. 2004
	9	8	31.1	3.89	25.4	9.3	55.6	Meza et al. 2004
	10	5	30.8	6.16	22.9	7.5	47.7	Meza et al. 2004

Finland	35	170	58.0^b^		19.0^b^	9.0^b^	67.0^b^	Kurttio et al. 1998
	9	< 1	5^b^		22.0^b^	9.0^b^	59.0^b^	Kurttio et al. 1998

Northeastern Taiwan	242	93	96.9	1.04	11.9	14.2	74.2	Hsueh et al. 2003

Taipei, Taiwan	313	10	25.5	2.55	5.5	7.9	86.6	Pu et al. 2007

Inner Mongolia, China	43	410	2,387.0	5.82	15.1	19.6	65.3	Pi et al. 2002
	10	20	263.0	13.15	27.0	15.2	57.8	Pi et al. 2002

Bangladesh	1,041	96	133.0^b^	1.39	15.8	12.6	70.7	Ahsan et al. 2007
	24	373	484.0	1.30	18.5	17.7	64.3	Chowdhury et al. 2003
	24	3	21.0	7.01	21.1	15.4	63.5	Chowdhury et al. 2003

a Median.

b Geometric mean.

## References

[b1-ehp-117-1860] Ahsan H, Chen Y, Kibriya MG, Slavkovich V, Parvez F, Jasmine F (2007). Arsenic metabolism, genetic susceptibility, and risk of premalignant skin lesions in Bangladesh. Cancer Epidemiol Biomarkers Prev.

[b2-ehp-117-1860] ATSDR (2000). Toxicological Profile for Arsenic.

[b3-ehp-117-1860] Buchet JP, Lauwerys R (1987). Study of factors influencing the *in vivo* methylation of inorganic arsenic in rats. Toxicol Appl Pharmacol.

[b4-ehp-117-1860] Buchet JP, Lauwerys R, Mahieu P, Geubel A (1982). Inorganic arsenic metabolism in man. Arch Toxicol Suppl.

[b5-ehp-117-1860] Buchet JP, Lauwerys R, Roels H (1981). Urinary excretion of inorganic arsenic and its metabolites after repeated ingestion of sodium metaarsenite by volunteers. Int Arch Occup Environ Health.

[b6-ehp-117-1860] Calderon RL, Hudgens E, Le XC, Schreinemachers D, Thomas DJ (1999). Excretion of arsenic in urine as a function of exposure to arsenic in drinking water. Environ Health Perspect.

[b7-ehp-117-1860] Chen CJ, Chen CW, Wu MM, Kuo TL (1992). Cancer potential in liver, lung, bladder and kidney due to ingested inorganic arsenic in drinking water. Br J Cancer.

[b8-ehp-117-1860] Chen CJ, Chuang YC, Lin TM, Wu HY (1985). Malignant neoplasms among residents of a blackfoot disease-endemic area in Taiwan: high-arsenic artesian well water and cancers. Cancer Res.

[b9-ehp-117-1860] Chen CJ, Hsueh YM, Lai MS, Shyu MP, Chen SY, Wu MM (1995). Increased prevalence of hypertension and long-term arsenic exposure. Hypertension.

[b10-ehp-117-1860] Chen Y, Factor-Litvak P, Howe GR, Graziano JH, Brandt-Rauf P, Parvez F (2007). Arsenic exposure from drinking water, dietary intakes of B vitamins and folate, and risk of high blood pressure in Bangladesh: a population-based, cross-sectional study. Am J Epidemiol.

[b11-ehp-117-1860] Chen YC, Guo YL, Su HJ, Hsueh YM, Smith TJ, Ryan LM (2003a). Arsenic methylation and skin cancer risk in southwestern Taiwan. J Occup Environ Med.

[b12-ehp-117-1860] Chen YC, Su HJ, Guo YL, Hsueh YM, Smith TJ, Ryan LM (2003b). Arsenic methylation and bladder cancer risk in Taiwan. Cancer Causes Control.

[b13-ehp-117-1860] Chowdhury UK, Rahman MM, Sengupta MK, Lodh D, Chanda CR, Roy S (2003). Pattern of excretion of arsenic compounds [arsenite, arsenate, MMA(V), DMA(V)] in urine of children compared to adults from an arsenic exposed area in Bangladesh. J Environ Sci Health Part A Toxic/Hazard Subst Environ Eng.

[b14-ehp-117-1860] Chung JS, Kalman DA, Moore LE, Kosnett MJ, Arroyo AP, Beeris M (2002). Family correlations of arsenic methylation patterns in children and parents exposed to high concentrations of arsenic in drinking water. Environ Health Perspect.

[b15-ehp-117-1860] Concha G, Nermell B, Vahter MV (1998). Metabolism of inorganic arsenic in children with chronic high arsenic exposure in northern Argentina. Environ Health Perspect.

[b16-ehp-117-1860] Concha G, Vogler G, Nermell B, Vahter M (2002). Intra-individual variation in the metabolism of inorganic arsenic. Int Arch Occup Environ Health.

[b17-ehp-117-1860] Coppin JF, Qu W, Waalkes MP (2008). Interplay between cellular methyl metabolism and adaptive efflux during oncogenic transformation from chronic arsenic exposure in human cells. J Biol Chem.

[b18-ehp-117-1860] Dewar WA, Lenihan JM (1956). A case of chronic arsenical poisoning; examination of tissue samples by activation analysis. Scott Med J.

[b19-ehp-117-1860] Fujihara J, Fujii Y, Agusa T, Kunito T, Yasuda T, Moritani T (2009). Ethnic differences in five intronic polymorphisms associated with arsenic metabolism within human arsenic (+3 oxidation state) methyltransferase (AS3MT) gene. Toxicol Appl Pharmacol.

[b20-ehp-117-1860] Fujihara J, Soejima M, Koda Y, Kunito T, Takeshita H (2008). Asian specific low mutation frequencies of the M287T polymorphism in the human arsenic (+3 oxidation state) methyltransferase (AS3MT) gene. Mutat Res.

[b21-ehp-117-1860] Gamble MV, Liu X, Ahsan H, Pilsner R, Ilievski V, Slavkovich V (2005). Folate, homocysteine, and arsenic metabolism in arsenic-exposed individuals in Bangladesh. Environ Health Perspect.

[b22-ehp-117-1860] Gonsebatt ME, Vega L, Salazar AM, Montero R, Guzman P, Blas J (1997). Cytogenetic effects in human exposure to arsenic. Mutat Res.

[b23-ehp-117-1860] Hernández A, Xamena N, Sekaran C, Tokunaga H, Sampayo-Reyes A, Quinteros D (2008). High arsenic metabolic efficiency in AS3MT287Thr allele carriers. Pharmacogenet Genomics.

[b24-ehp-117-1860] Hindmarsh JT (2002). Caveats in hair analysis in chronic arsenic poisoning. Clin Biochem.

[b25-ehp-117-1860] Hopenhayn-Rich C, Biggs ML, Kalman DA, Moore LE, Smith AH (1996a). Arsenic methylation patterns before and after changing from high to lower concentrations of arsenic in drinking water. Environ Health Perspect.

[b26-ehp-117-1860] Hopenhayn-Rich C, Biggs ML, Smith AH, Kalman DA, Moore LE (1996b). Methylation study of a population environmentally exposed to arsenic in drinking water. Environ Health Perspect.

[b27-ehp-117-1860] Hsueh YM, Chiou HY, Huang YL, Wu WL, Huang CC, Yang MH (1997). Serum beta-carotene level, arsenic methylation capability, and incidence of skin cancer. Cancer Epidemiol Biomarkers Prev.

[b28-ehp-117-1860] Hsueh YM, Hsu MK, Chiou HY, Yang MH, Huang CC, Chen CJ (2002). Urinary arsenic speciation in subjects with or without restriction from seafood dietary intake. Toxicol Lett.

[b29-ehp-117-1860] Hsueh YM, Huang YL, Huang CC, Wu WL, Chen HM, Yang MH (1998). Urinary levels of inorganic and organic arsenic metabolites among residents in an arseniasis-hyperendemic area in Taiwan. J Toxicol Environ Health A.

[b30-ehp-117-1860] Hsueh YM, Ko YF, Huang YK, Chen HW, Chiou HY, Huang YL (2003). Determinants of inorganic arsenic methylation capability among residents of the Lanyang Basin, Taiwan: arsenic and selenium exposure and alcohol consumption. Toxicol Lett.

[b31-ehp-117-1860] Huang YK, Huang YL, Hsueh YM, Yang MH, Wu MM, Chen SY (2008). Arsenic exposure, urinary arsenic speciation, and the incidence of urothelial carcinoma: a twelve-year follow-up study. Cancer Causes Control.

[b32-ehp-117-1860] Huang YK, Tseng CH, Huang YL, Yang MH, Chen CJ, Hsueh YM (2007). Arsenic methylation capability and hypertension risk in subjects living in arseniasis-hyperendemic areas in southwestern Taiwan. Toxicol Appl Pharmacol.

[b33-ehp-117-1860] Hughes MF, Kenyon EM, Edwards BC, Mitchell CT, Razo LM, Thomas DJ (2003). Accumulation and metabolism of arsenic in mice after repeated oral administration of arsenate. Toxicol Appl Pharmacol.

[b34-ehp-117-1860] IARC (International Agency for Research on Cancer) (1980). Some Metals and Metallic Compounds. IARC Monogr Eval Carcinog Risk Hum.

[b35-ehp-117-1860] Kenyon EM, Hughes MF, Adair BM, Highfill JH, Crecelius EA, Clewell HJ (2008). Tissue distribution and urinary excretion of inorganic arsenic and its methylated metabolites in C57BL6 mice following subchronic exposure to arsenate in drinking water. Toxicol Appl Pharmacol.

[b36-ehp-117-1860] Kuo TL (1964). Arsenic content of artesian well water in endemic area of chronic arsenic poisoning. Rep Inst Pathol Natl Taiwan Univ.

[b37-ehp-117-1860] Kurttio P, Komulainen H, Hakala E, Kahelin H, Pekkanen J (1998). Urinary excretion of arsenic species after exposure to arsenic present in drinking water. Arch Environ Contam Toxicol.

[b38-ehp-117-1860] Le XC, Cullen WR, Reimer KJ (1994). Human urinary arsenic excretion after one-time ingestion of seaweed, crab, and shrimp. Clin Chem.

[b39-ehp-117-1860] Mandal BK, Ogra Y, Suzuki KT (2001). Identification of dimethylarsinous and monomethylarsonous acids in human urine of the arsenic-affected areas in West Bengal, India. Chem Res Toxicol.

[b40-ehp-117-1860] Marafante E, Bertolero F, Edel J, Pietra R, Sabbioni E (1982). Intracellular interaction and biotransformation of arsenite in rats and rabbits. Sci Total Environ.

[b41-ehp-117-1860] Marnell LL, Garcia-Vargas GG, Chowdhury UK, Zakharyan RA, Walsh B, Avram MD (2003). Polymorphisms in the human monomethylarsonic acid (MMA V) reductase/hGSTO1 gene and changes in urinary arsenic profiles. Chem Res Toxicol.

[b42-ehp-117-1860] Meza MM, Kopplin MJ, Burgess JL, Gandolfi AJ (2004). Arsenic drinking water exposure and urinary excretion among adults in the Yaqui Valley, Sonora, Mexico. Environ Res.

[b43-ehp-117-1860] Mühlberg W, Platt D (1999). Age-dependent changes of the kidneys: pharmacological implications. Gerontology.

[b44-ehp-117-1860] Pi J, Yamauchi H, Kumagai Y, Sun G, Yoshida T, Aikawa H (2002). Evidence for induction of oxidative stress caused by chronic exposure of Chinese residents to arsenic contained in drinking water. Environ Health Perspect.

[b45-ehp-117-1860] Pu YS, Yang SM, Huang YK, Chung CJ, Huang SK, Chiu AW-H (2007). Urinary arsenic profile affects the risk of urothelial carcinoma even at low arsenic exposure. Toxicol Appl Pharmacol.

[b46-ehp-117-1860] Schläwicke Engström K, Broberg K, Concha G, Nermell B, Warholm M, Vahter M (2007). Genetic polymorphisms influencing arsenic metabolism: evidence from Argentina. Environ Health Perspect.

[b47-ehp-117-1860] Schläwicke Engström K, Nermell B, Concha G, Stromberg U, Vahter M, Broberg K (2009). Arsenic metabolism is influenced by polymorphisms in genes involved in one-carbon metabolism and reduction reactions. Mutat Res.

[b48-ehp-117-1860] Schmuck EM, Board PG, Whitbread AK, Tetlow N, Cavanaugh JA, Blackburn AC (2005). Characterization of the monomethylarsonate reductase and dehydroascorbate reductase activities of omega class glutathione transferase variants: implications for arsenic metabolism and the age-at-onset of Alzheimer’s and Parkinson’s diseases. Pharmacogenet Genomics.

[b49-ehp-117-1860] Steinmaus C, Bates MN, Yuan Y, Kalman D, Atallah R, Rey OA (2006). Arsenic methylation and bladder cancer risk in case-control studies in Argentina and the United States. J Occup Environ Med.

[b50-ehp-117-1860] Steinmaus C, Yuan Y, Kalman D, Atallah R, Smith AH (2005). Intraindividual variability in arsenic methylation in a U.S. population. Cancer Epidemiol Biomarkers Prev.

[b51-ehp-117-1860] Styblo M, Del Razo LM, Vega L, Germolec DR, LeCluyse EL, Hamilton GA (2000). Comparative toxicity of trivalent and pentavalent inorganic and methylated arsenicals in rat and human cells. Arch Toxicol.

[b52-ehp-117-1860] Thomas DJ, Styblo M, Lin S (2001). The cellular metabolism and systemic toxicity of arsenic. Toxicol Appl Pharmacol.

[b53-ehp-117-1860] Tseng CH, Huang YK, Huang YL, Chung CJ, Yang MH, Chen CJ (2005). Arsenic exposure, urinary arsenic speciation, and peripheral vascular disease in blackfoot disease-hyperendemic villages in Taiwan. Toxicol Appl Pharmacol.

[b54-ehp-117-1860] Vahter M (1999). Methylation of inorganic arsenic in different mammalian species and population groups. Sci Prog.

[b55-ehp-117-1860] Vahter M (2000). Genetic polymorphism in the biotransformation of inorganic arsenic and its role in toxicity. Toxicol Lett.

[b56-ehp-117-1860] Vahter ME (2007). Interactions between arsenic-induced toxicity and nutrition in early life. J Nutr.

[b57-ehp-117-1860] Vahter M, Concha G, Nermell B, Nilsson R, Dulout F, Natarajan AT (1995). A unique metabolism of inorganic arsenic in native Andean women. Eur J Pharmacol.

[b58-ehp-117-1860] Vahter M, Norin H (1980). Metabolism of 74As-labeled trivalent and pentavalent inorganic arsenic in mice. Environ Res.

[b59-ehp-117-1860] Valenzuela OL, Borja-Aburto VH, Garcia-Vargas GG, Cruz-Gonzalez MB, Garcia-Montalvo EA, Calderon-Aranda ES (2005). Urinary trivalent methylated arsenic species in a population chronically exposed to inorganic arsenic. Environ Health Perspect.

[b60-ehp-117-1860] Vega L, Styblo M, Patterson R, Cullen W, Wang C, Germolec D (2001). Differential effects of trivalent and pentavalent arsenicals on cell proliferation and cytokine secretion in normal human epidermal keratinocytes. Toxicol Appl Pharmacol.

[b61-ehp-117-1860] Wood TC, Salavaggione OE, Mukherjee B, Wang L, Klumpp AF, Thomae BA (2006). Human arsenic methyltransferase (AS3MT) pharmacogenetics: gene resequencing and functional genomics studies. J Biol Chem.

[b62-ehp-117-1860] Wu HY, Chen KP, Tseng WP, Hsu CL (1961). Epidemiologic studies on blackfoot disease: I. Prevalence and incidence of the disease by age, sex, occupation and geographical distribution. Mem College Med Natl Taiwan Univ.

[b63-ehp-117-1860] Wu MM, Kuo TL, Hwang YH, Chen CJ (1989). Dose-response relation between arsenic concentration in well water and mortality from cancers and vascular diseases. Am J Epidemiol.

[b64-ehp-117-1860] Yamauchi H, Yamamura Y (1985). Metabolism and excretion of orally administrated arsenic trioxide in the hamster. Toxicology.

[b65-ehp-117-1860] Yu RC, Hsu KH, Chen CJ, Froines JR (2000). Arsenic methylation capacity and skin cancer. Cancer Epidemiol Biomarkers Prev.

